# Supercontinuum generation in bulk solid-state material with bursts of femtosecond laser pulses

**DOI:** 10.1038/s41598-024-57928-9

**Published:** 2024-03-25

**Authors:** B. Momgaudis, V. Marčiulionytė, V. Jukna, G. Tamošauskas, M. Barkauskas, A. Dubietis

**Affiliations:** 1 Laser Research Center, Vilnius University, Saulėtekio Avenue 10, 10223 Vilnius, Lithuania; 2 Light Conversion Ltd., Keramiku̧ 2B, 10233 Vilnius, Lithuania

**Keywords:** Optics and photonics, Supercontinuum generation

## Abstract

We report on experimental and numerical investigation of burst-mode supercontinuum generation in sapphire crystal. The experiments were performed using bursts consisting of two 190 fs, 1030 nm pulses with intra-burst repetition rates of 62.5 MHz and 2.5 GHz from an amplified 1 MHz Yb:KGW laser and revealed higher filamentation and supercontinuum generation threshold for the second pulse in the burst, which increases with the increase of intra-burst repetition rate. The experimental results were quantitatively reproduced numerically, using a developed model, which accounted for altered material response due to residual excitations remaining after propagation of the first pulse. The simulation results unveiled that residual free electron plasma and self-trapped excitons contribute to elevated densities of free electron plasma generated by the second pulse in the burst and so stronger plasma defocusing, significantly affecting its nonlinear propagation dynamics. The presented results identify the fundamental and practical issues for supercontinuum generation in solid-state materials using femtosecond pulse bursts with very high intra-burst repetition rates, which may also apply to the case of single pulses at very high repetition rate, where residual material excitations become relevant and should be accounted for.

## Introduction

Burst trains of femtosecond laser pulses emerge as a powerful tool for laser material processing, offering a variety of technological innovations in micromachining of metals^1–5^, glasses^6–12^, semiconductors^13–15^, polymers^16^ and biomaterials^17^. Burst-mode two-photon polymerization was considered to deliver microstructures with smaller feature size^18^, while irradiation of solid targets with femtosecond pulse bursts was demonstrated to significantly increase of the hard X-ray yield^19^. For what concerns laser wavelength conversion processes, generation second and fourth harmonics with femtosecond pulse bursts was demonstrated^20^. Burst-mode pumping was shown beneficial for increasing the pump-to-idler conversion efficiency in long-picosecond mid-infrared optical parametric oscillator^21^. In the femtosecond regime, burst-mode operation allowed efficient average power scaling of femtosecond optical parametric oscillators^22,23^ and amplifiers^24,25^, as well as optical parametric chirped pulse amplifiers (OPCPA)^26,27^ and spectral broadening-based extracavity post-compression schemes^28,29^, which deliver few optical cycle pulses.

Yet, only a few attempts were carried out to study filamentation phenomena in transparent materials using femtosecond pulse bursts. Burst-mode filamentation in atmospheric air enabled control and tailoring of filamentation dynamics^30^ and production of extended conductive channels via filament stitching^31^, which are important for long-distance applications. Time-resolved dynamics of burst-mode filamentation-assisted micromachining of glasses revealed build-up of heat accumulation^6^, brighter and longer-lasting filament luminescence tracks^8^, demonstrating stronger laser-matter interaction and more prominent morphological changes of the solid-state material compared to a single pulse exposure.

Supercontinuum (SC) generation is one of the most outstanding outcomes of femtosecond filamentation in solid-state materials, serving as a broadband seed source for ultrafast optical parametric amplifers^32^ and OPCPA^33^. In contrast to laser material processing-oriented applications, burst-mode SC generation requires robust, durable and modification-free performance of the nonlinear material. However, so far, burst-mode SC generation for seeding an OPA was demonstrated just at relatively low (188 kHz) intra-burst repetition rate^24^, and to the best of our knowledge, burst-mode SC generation in bulk solid-state materials with very high (tens of MHz to a few GHz) intra-burst repetition rates was not studied so far. In this Paper we present experimental and numerical investigation of burst-mode filamentation and SC generation in sapphire crystal, demonstrating that residual material excitations, such as free electron plasma and self-trapped excitons remaining after propagation of the preceding pulse, alter filamentation and SC generation dynamics of subsequent pulses, which is especially evident in the case of very high, GHz intra-burst repetition rates.

## Experimental observations

The experiments were performed with 190 fs, 1030 nm pulses from an amplified 80 W Yb:KGW laser (Carbide, Light Conversion Ltd.). The laser operated at 1 MHz repetition rate in either single-pulse or burst-mode, with intra-burst repetition rates of 62.5 MHz and 2.5 GHz, which correspond to a time delay between adjacent pulses of 16 ns and 400 ps, respectively. The laser enabled operation with an adjustable number (2–10) of pulses per burst, however in the present case, for the sake of simplicity we restricted ourselves to just two pulses per burst, which allowed to clearly observe experimentally and reproduce numerically the relevant burst-induced effects on beam filamentation and SC generation.

A typical SC generation geometry was used, where the input beam with a diameter of 4.6 mm (at the 1/e^2^ intensity level) was focused with a fused silica lens ($f=+100$ mm) onto the front face of uncoated and undoped 5-mm-long sapphire sample (EKSMA Optics). The spectral measurements were performed by varying the input pulse energy with an attenuator consisting of a half-wave plate and thin-film polarizer and coupling the axial part of the output radiation into a slit of spectrometer (AvaSpec-3648, Avantes) with a detection range from 200 to 1100 nm, so recording only the short wavelength side of the SC spectrum. The dynamic range of spectral measurements was increased by attenuating the most intense part of the SC radiation around the pump wavelength with a short-pass filter. The measured spectra thereafter were corrected accounting for the filter transmission function and the sensitivity function of the spectrometer.

**Figure 1:**
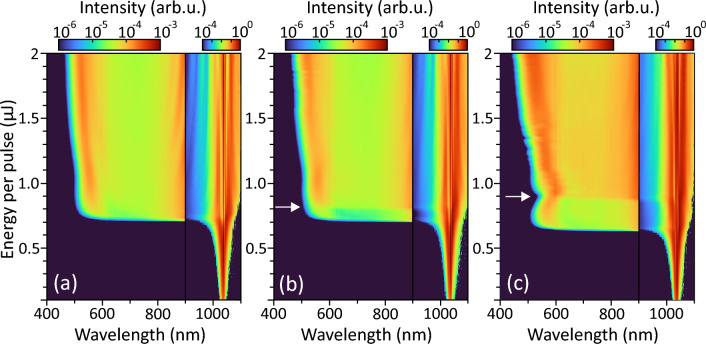
The dynamics of spectral broadening in sapphire versus the pump pulse energy measured in (**a**) single-pulse mode, and in burst-mode containing 2 pulses per burst with intra-burst repetition rates of (**b**) 62.5 MHz, (**c**) 2.5 GHz. Note different intensity scales in the wavelength ranges of $400-900$ nm and $900-1100$ nm, which were used for a better visibility of relevant spectral features. The arrows in (**b**) and (**c**) mark the threshold energies for the SC generation by the second pulse in the burst.

First of all, the spectral measurements were performed with a single pump pulse at 1 MHz laser repetition rate, which served as a reference for the burst-mode measurements. The measured dynamics of spectral broadening in a single-pulse mode is presented in Fig. 1a, showing an explosive broadening of the spectrum at the pump pulse energy of 710 nJ, which was defined as the threshold energy for SC generation. No significant spectral changes were observed with further increase of the pump pulse energy, showing fairly constant cut-off at $\sim 500$ nm and smooth spectral shape. Figure 1b shows the dynamics of spectral broadening measured in the burst-mode, containing 2 pulses per burst with intra-burst repetition rate of 62.5 MHz. An explosive spectral broadening is observed with the same energy of the pump pulse (here evaluated as an energy per pulse) as in the above case. However, in the present case, the SC is generated only by the first pulse, while a slightly higher energy (800 nJ) is required to produce SC by both pulses in the burst, as justified by an abrupt twofold increase of the SC spectral intensity. Even more distinctive picture of step-wise SC generation dynamics was recorded in the burst-mode with intra-burst repetition rate of 2.5 GHz, as illustrated in Fig. 1c. Firstly, the measured spectral dynamics revealed a slightly lower (650 nJ) threshold energy for SC generation with the first pulse in the burst, which we attribute to the possible influence of thermal effects due to high (1 MHz) repetition rate of the laser, locally affecting the condition of beam focusing. Secondly, and more importantly, the pump pulse energy has to be increased considerably, up to 900 nJ, to observe the SC generation by both pulses in the burst.

## The numerical simulations

To better understand the fundamental processes behind the burst-mode filamentation and SC generation, we developed the numerical model which enabled a detailed analysis of light-matter interaction considering the propagation of several consecutive laser pulses with an account for material response due to residual excitations remaining after propagation of preceding pulse. The laser pulse propagation through the nonlinear material was modelled with the use of the modified nonlinear Schroedinger equation, which accounted for nonparaxial diffraction, full dispersion equation of the material, optical Kerr effect, multiphoton absorption and free electron plasma^34^. The model of material response accounted for plasma generation resulting from multiphoton absorption, i.e. eight-photon absorption, in the present case, assuming the photon energy of the incident laser pulse of 1.2 eV (the laser wavelength 1030 nm) and the bandgap of sapphire $U_e=9$ eV, and avalanche ionization. Formation of transient states within the band gap, such as self-trapped excitons (STEs)^35^ with recombination and binding energies of 7.5 eV and 1.5 eV, respectively, was also taken into consideration. The nonlinear absorption cross-sections of each transition were evaluated using Keldysh theory. The full description of the numerical model is provided in the Methods section.

The simulation procedure for the excited state populations was performed as follows. First, the excited state populations were calculated simultaneously with the propagation of the first pulse, within a time frame equal to five times the pulse duration. Thereafter the relatively slow relaxation processes (relaxation of electron population in the conduction band, formation and decay of STEs) within the time interval between adjacent pulses were simulated separately with much greater time step. These residual populations defined the altered state of material at the moment of the second laser pulse arrival. More specifically, the residual spatial distributions of the electron and STE densities influences the nonlinear absorption and linear pulse propagation properties due to refractive index change, which was accounted via Drude-Lorentz model formalism.

**Figure 2:**
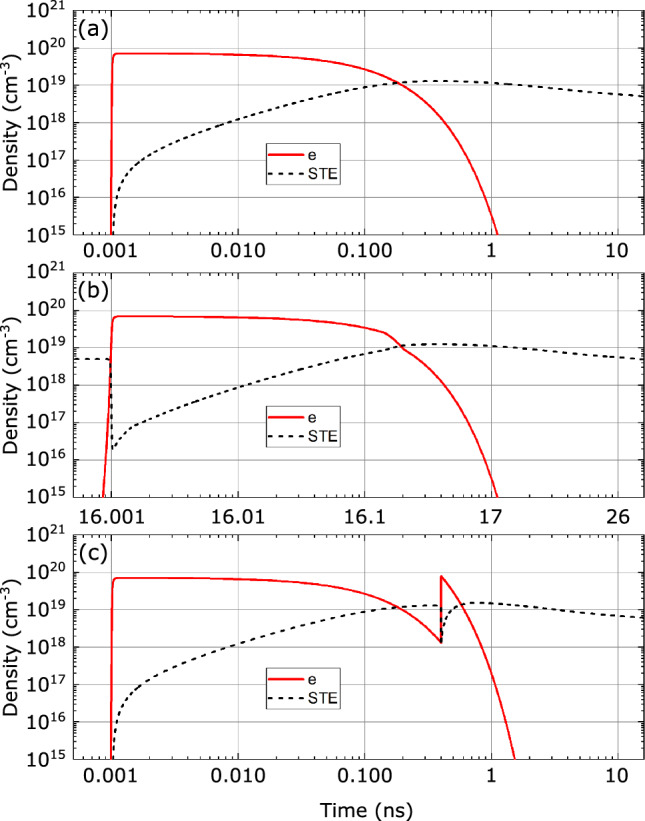
Free electron plasma (solid curves) and STE (dashed curves) dynamics after passage of (**a**) first pulse, (**b**) second pulse delayed by 16 ns, (**c**) second pulse delayed by 400 ps. The pulse energy in all the cases is $0.83~\upmu$J. See text for details.

Figure 2 presents the simulated dynamics of free electron plasma and STE (the sum of all three sub-states) densities in the beam center at a fixed propagation distance, $z=2.06$ mm, which corresponded to the position of nonlinear focus (where intensity is the highest) of the first pulse, and which slightly varied for the second pulse due to presence of residual free electrons and STEs, which locally induce either negative or positive contributions to the refractive index, respectively. Figure 2a shows the time evolutions of free electron plasma and STE densities induced by the propagation of the first laser pulse with an energy of $0.83~\upmu$J, that is slightly above the experimentally established threshold energy for SC generation. The free electron plasma due to transitions from valence to conduction band rises rapidly (within the time interval approximately equal to pulse duration) reaching the maximum density of $7.2\times 10^{-19}$ cm^-3^ and then decays via radiative (fluorescence) and nonradiative (excitation of phonons and formation of STEs) processes with decay time constant of $\tau _{r_e}=100$ ps^36^. The build up time of STEs was taken equivalent to the relaxation time of electrons in the conduction band. The density of STEs reaches its peak value of $1.1\times 10^{-19}$ cm^-3^ at approximately 380 ps after passage of the laser pulse, and thereafter decays with characteristic relaxation times of 2, 23 and 200 ns^35^. It was assumed that 20% of electrons in the conduction band form STEs upon relaxation, and the STE population distributions within the sub-states corresponding to these decay constants were taken as 2:1:1, respectively.

Figure 2b shows the simulated dynamics of free electron plasma and STE densities after passage of the second pulse in the burst, which is delayed by 16 ns (note a different timescale of a graph). In fact, the second pulse finds only a negligible residual concentration of free electrons, but still a notable residual density of STEs ($5.4\times 10^{-18}$ cm^-3^). At the moment of the second pulse arrival, there is an abrupt drop of STE density due to optically-induced dissociation of STEs via two-photon absorption, contributing to a slightly higher density ($8.4\times 10^{-19}$ cm^-3^) of free electron plasma in the wake of the second pulse. Thereafter the dynamics of STE density follows essentially the same trend as in the case of the first pulse.

Figure 2c shows the simulated dynamics of free electron plasma and STE densities after passage of the second pulse in the burst, which is delayed by 400 ps. In this case, the second pulse encounters a material with still appreciable concentrations of both, free electrons and STEs ($1.3\times 10^{-18}$ cm^-3^ and $1.3\times 10^{-19}$ cm^-3^, respectively). The combined effect of residual free electrons that provide initial population for faster onset of avalanche ionization and optically-induced dissociation of residual STEs yields a maximum plasma density of $9.1\times 10^{-19}$ cm^-3^, which is larger by almost 30% than the plasma density produced by the first pulse. Also note an abrupt drop of STE density due to optically-induced dissociation that is followed by the build-up of STE states as free electron plasma decays, reaching the maximum density of $1.5\times 10^{-19}$ cm^-3^.

**Figure 3:**
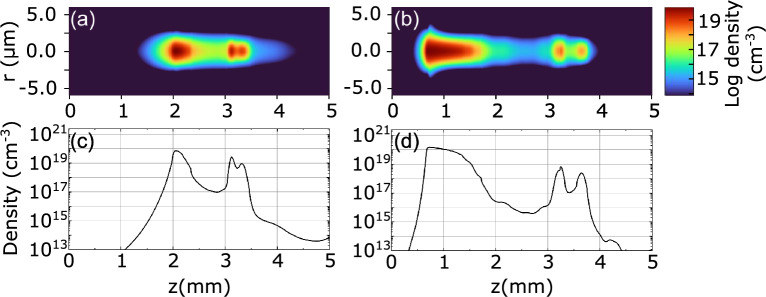
(**a**), (**b**) radial and (**c**), (**d**) axial (at the center of the beam) distributions of free electron plasma along the beam propagation path produced by nonlinear propagation of the first pulse with energies of $0.83~\upmu$J and $1.28~\upmu$J, respectively.

The free electron plasma and STE dynamics in the case of pulses with higher energy, $1.28~\upmu$J, where SC generation by both pulses in the burst regardless of their temporal separation was observed experimentally, are not shown, since they follow essentially identical trends, as illustrated in Fig. 2. However, the main differences occur in the longitudinal (i.e. along the beam propagation path) distributions of free electron plasma, which drives the nonlinear propagation dynamics of the pulse. Figure 3 compares the calculated radial and axial (at the center of the beam) distributions of free electron plasma densities along the beam propagation path induced by the propagation of laser pulse with energies of $0.83~\upmu$J and $1.28~\upmu$J, which are very illustrative to general understanding of nonlinear propagation dynamics, and which are explained in detail in the following section. Also note that the difference of maximum achievable free electron plasma densities with lower and higher energy pulses is small due to the intensity clamping effect.

## Results and discussion

**Figure 4:**
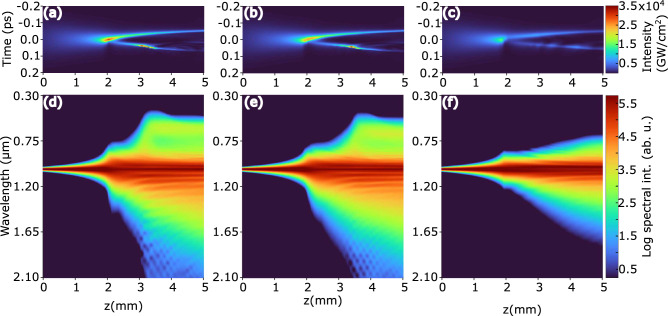
(**a**) Temporal dynamics of the first laser pulse in “fresh” sapphire crystal. The input pulse duration is 190 fs, energy is $0.83~\upmu$J, and *z* denotes the propagation distance. Temporal dynamics of identical second laser pulse, which is delayed by (**b**) 16 ns and (**c**) 400 ps. Panels (**d**), (**e**) and (**f**) display the corresponding spectral dynamics.

To start with, we numerically simulated the nonlinear propagation of the first laser pulse with an energy of $0.83~\upmu$J in a “fresh”, i.e. unaltered nonlinear material (sapphire crystal), whose temporal and spectral dynamics at the center of the beam versus the propagation distance *z* are shown in Fig. 4a. We note that in the present case, due to relatively long duration (190 fs) of the driving pulse, the SC generation scenario is rather specific (showing step-wise spectral broadening versus the propagation distance *z*)^37^ and is different from that of SC generation with $\sim 100$ fs and shorter pulses in the nonlinear material with normal group velocity dispersion, where spectral broadening takes place in an explosive manner, as being solely governed by the pulse splitting at the nonlinear focus of the beam and subsequent self-steeping of the sub-pulses, see e.g.^38^.

More precisely, free electron plasma produced by the leading front of the driving pulse (see the plasma density peak at $z\approx 2.1$ mm in Fig. 3a, c, which denotes the nonlinear focus of the beam), efficiently absorbs and defocuses the rear part (the descending front) of the pulse. As a result, the pulse at the beam center experiences a considerable reduction of its temporal width and its intensity peak shifts toward the forefront of the pulse. Such plasma-induced pulse shortening does not produce SC and results only in moderate spectral broadening beyond the nonlinear focus, as illustrated in Fig. 4d. Meanwhile a considerable fraction of energy contained in the center and rear part of the pulse is being pushed out of the propagation axis, developing a specific ring-shaped spatial intensity distribution^39^. With further propagation, the pulse intensity starts decreasing due to combined action of nonlinear losses, plasma defocusing and group velocity dispersion-induced temporal broadening, so the plasma generation as well as it‘s induced defocusing decline, and the peripheral part is able to replenish the pulse at the beam center. The replenishment position along the propagation axis *z* is defined by the peak density and geometry (width and length) of the plasma channel. Because of such dramatic spatiotemporal reshaping, the replenished pulse has notably shorter duration than the incident pulse, so despite the fact that the replenished pulse contains only a fraction of the incident pulse energy, its power is above the critical power for self-focusing. As a result, a short replenished pulse self-focuses at $z\approx 3.1$ mm, where it experiences pulse splitting and dramatic self-steepening of the trailing sub-pulse at $z\approx 3.3$ mm, see the corresponding plasma peaks in Fig. 3a, c, that results in SC generation in accordance to general SC generation scenario^38^, as justified by the corresponding spectral dynamics shown in Fig. 4d. It has to be noted that the pulse splitting is very asymmetric in terms of intensity: producing very intense trailing sub-pulse, and faint, barely noticeable leading sub-pulse, whose self-steepenings are responsible for the generation of blue-shifted and red-shifted components of the SC spectrum, respectively. The regular spectral fringes, which are particularly distinct on the long wavelength side, appear due to the interference between plasma-shortened and spectrally broadened pulse at the forefront and SC-producing split sub-pulses.

Figure 4b shows the temporal dynamics of the second laser pulse delayed by 16 ns, which finds slightly altered nonlinear material featuring notable density of STEs generated by the propagation of the first pulse (see Fig. 2b). However, this has only very minor effect on the second pulse propagation, which undergoes almost identical spatio-temporal transformations, with splitting of the replenished pulse and SC generation occurring at a slightly longer ($z\approx 3.5$ mm) propagation distance, see Fig. 4e. This may explain the experimental evidence (Fig. 1b) why a slightly larger energy of the second pulse in the 62.5 MHz burst is required for SC generation to occur.

A different picture emerges when the second laser pulse is delayed by 400 ps, as illustrated in Fig. 4c. In this case, the second pulse encounters a material with still considerable densities of both, free electron plasma and STEs, as shown in Fig. 2c. As a result, the pulse experiences additional energy losses due to plasma and STE absorption and stronger plasma defocusing due to its larger density. Although its temporal dynamics visually follow the initial stage of plasma-induced pulse reshaping as described above, the replenished pulse never acquires enough power to self-focus. Consequently, no splitting of the replenished pulse occurs and no SC is produced. Only gradual spectral broadening that is limited to the near-infrared range takes place due to continuous plasma-induced pulse reshaping, as shown in Fig. 4f.

**Figure 5:**
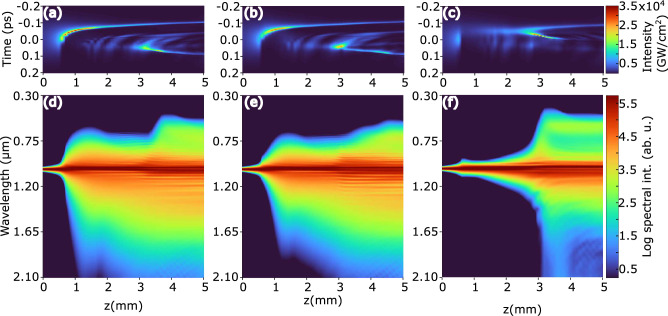
Temporal dynamics of (**a**) the first laser pulse with energy of $1.28~\upmu$J and the second pulse delayed by (**b**) 16 ns and (**c**) 400 ps. Panels (**d**), (**e**) and (**f**) show the corresponding spectral dynamics.

Figure 5 shows the numerically simulated temporal and spectral dynamics of higher energy ($1.28~\upmu$J) input pulses. The temporal dynamics of the first pulse is depicted in Fig. 5a. Compared to the lower input pulse energy case (Fig. 4a), here the nonlinear focus is located much closer to the entrance face of the nonlinear material (at $z\approx 0.5$ mm), and the pulse experiences a dramatic plasma-induced reduction of its width that results in significant spectral broadening, which is especially pronounced on the long wavelength side, see Fig. 5d. At the same time, an extended plasma channel ($\sim$1 mm-long, with almost uniform free electron density of $\sim 10^{-20}$ cm^-3^, see Fig. 3b, d) is produced in the pulse wake, so the replenished pulse at the beam center emerges only after $z\approx 2.8$ mm of propagation. The replenished pulse thereafter self-focuses and undergoes pulse splitting $z\approx 3.1$ mm, while explosive broadening of the spectrum towards the short wavelength side induced by self-steepening of the trailing sub-pulse takes place at $z\approx 3.7$ mm, see Fig. 5d. In the present case, the overall nonlinear dynamics resembles complex temporal evolution of a picosecond laser pulse undergoing filamentation to a great extent^39^.

The temporal dynamics of the second laser pulse that is delayed by 16 ns is depicted in Fig. 5b. Although the general temporal dynamics of the second pulse is very similar to that of the first pulse in the burst, after splitting of replenished pulse, the trailing sub-pulse experiences slower self-steepening, and so produces rather gradual than explosive spectral broadenings towards short wavelength side, see Fig. 5e.

The temporal dynamics of the second laser pulse that is delayed by 400 ps is illustrated in Fig. 5c, demonstrating its completely different temporal behavior. In this case, residual free electrons and STEs facilitate the generation of higher density free electron plasma via faster onset of avalanche ionization and optically-induced dissociation of STEs, whose radial distribution mimics the distribution of free electrons shown in Fig. 3b. As a result, the pulse experiences very strong defocusing as a whole, without producing a characteristic plasma-shortened fragment. The replenished pulse gradually emerges at the temporal position that is shifted to the forefront, in the time frame of the input pulse, until self-focuses and splits at $z\sim 2.5$ mm, yielding very broad SC spectrum, whose evolution versus the propagation distance is shown in Fig. 5f.

It is possible to simulate the propagation of many successive laser pulses in the burst, putting this algorithm in a loop. In the present set of simulations, the model of material response neglected formation of F point defects^40^, such as electrons trapped in oxygen vacancies^41,42^ and oxygen bi-vacancies^43^, which would occur due to non-radiative decay of STEs^44^. Indeed, these processes are assumed to be very slow on the short time scale where the propagation of small number of laser pulses is considered. However, formation of F point defects may become detrimental if the material is continuously exposed to a very large number of pulses, eventually leading to permanent structural modifications, e.g. nanograting formation^45^. Similar considerations apply for what concerns other slow processes, such as local heating, heat diffusion and pressure wave propagation, which would certainly play a role in the case of many pulses in the burst, as well as in the case of continuous irradiation of nonlinear material by single laser pulses at very high repetition rate.

## Conclusions and outlook

In conclusion, we investigated experimentally and numerically burst-mode filamentation and SC generation in solid-state material (sapphire crystal) with bursts containing two identical femtosecond pulses and with intra-burst repetition rates of 62.5 MHz and 2.5 GHz, which correspond to a time delay between adjacent pulses of 16 ns and 400 ps, respectively. The measured spectral dynamics versus pulse energy demonstrated an increase of SC generation threshold for the second pulse in the burst, and this increase was found significant in the case of 2.5 GHz burst.

In order to explain the experimental observations, we developed a numerical model, which accounted for residual material excitations, such as electrons in the conduction band and STEs. The numerical simulations reproduced the experimental findings very well, providing the insights into temporal and spectral dynamics of individual pulses in the burst. It is demonstrated that residual free electron plasma and STEs from the first pulse contribute to elevated densities of free electron plasma generated by the successive pulse thus substantially altering dynamics of its nonlinear propagation and spectral broadening. More specifically, the residual free electron plasma provides initial population for faster onset of avalanche ionization, while optically-induced dissociation of STEs contributes to an increase of electron density in the conduction band, resulting in stronger plasma defocusing experienced by the second pulse in the burst. The investigated example of sapphire as the nonlinear material, suggests that in the case of 2.5 GHz burst, the nonlinear dynamics of successive pulse is affected by both effects, whereas in the case of 62 MHz burst, the nonlinear dynamics of successive pulse is affected mainly by optically-induced dissociation of residual STEs. The numerical simulations also unveiled how the temporal dynamics of the pulse, and the second pulse in the burst, in particular, change when the pulse energy increases, which could not be retrieved by simple means experimentally. These results are in contrast with burst-mode SC generation in photonic crystal fiber^46^, where the pulse intensities are well below that produce multiphoton absorption and ionization effects as well as formation of STEs, and thus only a weak dependence of SC characteristics on temporal delay between the driving pulses was observed.

The numerical model provides a useful framework for better understanding of burst-mode filamentation in transparent solid-state materials and identifies potential practical issues arising from residual material excitations. The model could be elaborated further by including the processes with slow build-up times, such as local heating, heat diffusion, pressure wave propagation and eventually, formation of F centers, which all become relevant on a long time scale considering either propagation of many successive laser pulses in the burst or irradiation of the nonlinear material by single laser pulses at very high, sub-GHz and multi-GHz repetition rates. In that regard, our study also suggests that the lifetimes of transient excitations and carrier dynamics are of utmost importance regarding robust and durable operation of the nonlinear material in such adverse pumping conditions.

## Methods

The numerical simulations were performed by solving the unidirectional nonlinear propagation equation for the pulse envelope in the spectral domain^34^:

1 $$\begin{aligned} \frac{\partial S(\Omega ,k_{\perp })}{\partial z}+iD(\Omega ,k_{\perp })=S_N(\Omega ,k_{\perp }), \end{aligned}$$

where *z* is the propagation coordinate, $\Omega =\omega -\omega _0$ is the frequency shift from the carrier frequency$\omega _0$ and $k_{\perp }$ is the transverse wave number. The function $D(\Omega ,k_{\perp })$ describes the linear propagation of light and accounts for diffraction and dispersion of the wave packet (ultrashort-pulsed laser beam) in nonparaxial approximation:

2 $$\begin{aligned} D(\Omega ,k_{\perp })=\sqrt{k(\omega _0+\Omega )^2-k_{\perp }^2}-k_0-\frac{\Omega }{v_g}, \end{aligned}$$

where $v_g=\frac{\partial \omega }{\partial k}|_{\omega _0}$ is the group velocity, $k(\omega )= \frac{n(\omega )\omega }{c}$ describes the dispersion relation and $k_0=k(\omega _0)$. The complex pulse envelope *A*(*t*, *r*), where *t* and *r* are the time and radial coordinates, respectively, was calculated by applying Hankel-Fourier transform:

3 $$\begin{aligned} A(t,r)=\int _{-\infty }^{+\infty }\int _{0}^{+\infty }S(\Omega ,k_{\perp })e^{i\Omega t}J_0(k_{\perp }r) k_{\perp } dk_{\perp }\frac{d\Omega }{2\pi }, \end{aligned}$$

where $J_0$ is a zeroth-order Bessel function. The term on the right hand side of Eq. (1) accounts for nonlinear pulse propagation and is expressed as:

4 $$\begin{aligned} S_N(\Omega ,k_{\perp })=\int _{-\infty }^{+\infty }\int _{0}^{+\infty }N(t,r)e^{-i\Omega t}J_0(k_{\perp }r)rdrdt. \end{aligned}$$

The nonlinear term *N*(*t*, *r*) is defined as:

5 $$\begin{aligned} N(t,r)=\frac{i\omega _0n_2}{c}|A|^2A-\frac{\beta _e^{K_e}}{2}|A|^{2K_e-2}A-\frac{\beta _{STE}^{K_{STE}}}{2}|A|^{2K_{STE}-2}A-\frac{\sigma _e}{2}(1+i\omega _0\tau _c)\rho _e A-i\frac{\sigma _{STE}}{2}\rho _{STE}A. \end{aligned}$$

The first term in Eq. (5) refers to optical Kerr effect, where $n_2=2.8\times 10^{-16}$ cm^2^/W is the nonlinear refractive index. The second and third terms refer to the multi-photon absorption from the contributing states where $\beta$ stands for the absorption cross section, *K* denotes the multiphoton absorption order, and $\rho$ is the population density, while subscripts *e* and *STE* stand for electrons and STEs, respectively. The fourth term describes the contribution from the avalanche ionization estimated using Drude model, with the coefficient $\sigma _e$ given by:

6 $$\begin{aligned} \sigma _e=\frac{e^2\tau _c}{cn_0\epsilon _0m(1+\omega ^2_0\tau ^2_c)}, \end{aligned}$$

where $\tau _c=1.5$ fs is the electron collision time, $\epsilon _0$ is the vacuum permittivity, and *e* and *m* denote the electron charge and reduced mass, respectively. The fifth term describes the phase retardation induced by STEs via Lorentz model according to^47^:

7 $$\begin{aligned} \sigma _{STE}=\frac{\omega _0n(\omega _0)\omega ^2_{STE,crit}(\omega ^2_{STE}-\omega ^2_0)}{c((\omega ^2_{STE}-\omega ^2_0)+(\frac{\omega _0}{\tau _c})^2)}, \end{aligned}$$

where $\omega _{STE}$ is the resonant frequency of STEs and $\omega _{STE,crit}=\sqrt{\frac{e^2}{m\epsilon _0}}$.

The material excitation was calculated using rate equations for both electron and STE populations, taking into account the three characteristic relaxation times of the latter. The rate equation for electrons reads as

8 $$\begin{aligned} \frac{\partial \rho _e}{\partial t}=(\rho _n-\rho _e )W_{Mp_e}+\rho _eW_{Av}+\rho _{STE}W_{Mp_{STE}}-\rho _eW_{r_e}, \end{aligned}$$

where $\rho _n=2.1\times 10^{22}$ cm^-3^ is the density of bound electrons, $W_{Mp_e}=\frac{\beta _e}{K_e\hbar \omega _0}|A|^{2K_e}$ and $W_{Mp_{STE}}=\frac{\beta _{STE}}{K_{STE}\hbar \omega _0}|A|^{2K_{STE}}$ are the multiphoton ionization rates obtained using Keldysh formulation for electrons and STEs respectively, $W_{Av}=\sigma _e|A|^2/U_e$ is the avalanche ionization rate, and $W_{r_e}=1/\tau _{r_e}$ is the electron recombination rate. The simplified rate equation for STEs reads as

9 $$\begin{aligned} \frac{\partial \rho _{STE}}{\partial t}=-\rho _{STE}W_{Mp_{STE}}-\rho _{STE}W_{r_{STE}}+\rho _eW_{r_e}\eta _{STE}, \end{aligned}$$

where $\eta _{STE}$ is the exciton trapping efficiency, which was assumed to be 20%. The rate equations were solved simultaneously using sixth order Runge-Kutta method.

## Data Availability

The datasets used and/or analysed during the current study available from the corresponding author on reasonable request.
